# Predicting body mass index in early childhood using data from the first 1000 days

**DOI:** 10.1038/s41598-023-35935-6

**Published:** 2023-05-31

**Authors:** Erika R. Cheng, Ahmet Yahya Cengiz, Zina Ben Miled

**Affiliations:** 1 Division of Children’s Health Services Research, Department of Pediatrics, Indiana University School of Medicine, 410 W. 10th Street, Indianapolis, IN 46220 USA; 2 Department of Computer Science, Purdue School of Science, IUPUI, Indianapolis, IN USA; 3 Department of Electrical and Computer Engineering, School of Engineering and Technology, Indiana University Purdue University at Indianapolis, Indianapolis, IN USA; 4 Regenstrief Institute, Inc., Indianapolis, IN USA

**Keywords:** Health care, Risk factors

## Abstract

Few existing efforts to predict childhood obesity have included risk factors across the prenatal and early infancy periods, despite evidence that the first 1000 days is critical for obesity prevention. In this study, we employed machine learning techniques to understand the influence of factors in the first 1000 days on body mass index (BMI) values during childhood. We used LASSO regression to identify 13 features in addition to historical weight, height, and BMI that were relevant to childhood obesity. We then developed prediction models based on support vector regression with fivefold cross validation, estimating BMI for three time periods: 30–36 (N = 4204), 36–42 (N = 4130), and 42–48 (N = 2880) months. Our models were developed using 80% of the patients from each period. When tested on the remaining 20% of the patients, the models predicted children’s BMI with high accuracy (mean average error [standard deviation] = 0.96[0.02] at 30–36 months, 0.98 [0.03] at 36–42 months, and 1.00 [0.02] at 42–48 months) and can be used to support clinical and public health efforts focused on obesity prevention in early life.

## Introduction

The prevalence of overweight and obesity in the United States has increased dramatically during the past 40 years^1^. As of 2016, 39.8% of US adults and 20.6% of US adolescents had obesity, with a significantly higher prevalence among underserved populations^2^. Furthermore, while previously uncommon in young children, overweight and obesity now affect over 40 million children under the age of 5, an epidemic that is apparent worldwide^3,4^. In children, early obesity and excess weight gain not only predict later obesity and cardio-metabolic risk, but also serious morbidity within childhood^5–12^. Unfortunately, once obesity is established it is likely to persist^13–15^ and notoriously difficult to treat^16–19^. In light of these facts, there has been increasing focus on prevention as holding the most promise for addressing the obesity epidemic^20^.

The “first 1000 days” from conception until the end of the second year of life are recognized as a critical period for obesity prevention as this time frame offers both greater developmental plasticity and opportunity to impact obesogenic behaviors before they are established^21^. Identifying children at a young age who carry the greatest risk for obesity could therefore significantly improve prevention efforts^22^. A number of potentially modifiable risk factors during this timeframe have been identified, including higher maternal pre-pregnancy body mass index (BMI), maternal excess gestational weight gain, high infant birth weight, low socioeconomic status, and neighborhood-level factors (e.g., food availability, crime)^23^. But little is known about the predictive performance of such factors when considered jointly, as few existing population-based datasets contain socio-demographic data alongside measured heights and weights across pregnancy and early childhood, and birth cohorts do not always capture maternal data.

Machine learning (ML) is increasingly recognized as useful for preventive care^24^ and has the potential to identify patients with increased risk, enabling early intervention. One benefit to ML techniques is the ability to examine how risk factors across multiple life course periods (e.g., prenatal and early life) and settings (e.g., home and neighborhood) interact to affect childhood obesity risk. Although a number of studies have applied ML to predict obesity in early childhood^25,26^, most are derived from non-US cohorts, focus on identifying predictors of obesity after birth, and/or rely exclusively on clinical data. Gaining a better delineation of intergenerational and social predictors of obesity risk in the first 1000 days is important for the prevention of obesity and could have downstream effects on mitigating the obesity epidemic as a whole.

We used ML technology to understand the influence of factors in the first 1000 days on BMI values during childhood and to develop a prediction model that can be applied during infancy for the early identification of obesity risk.

## Methods

**Figure 1:**
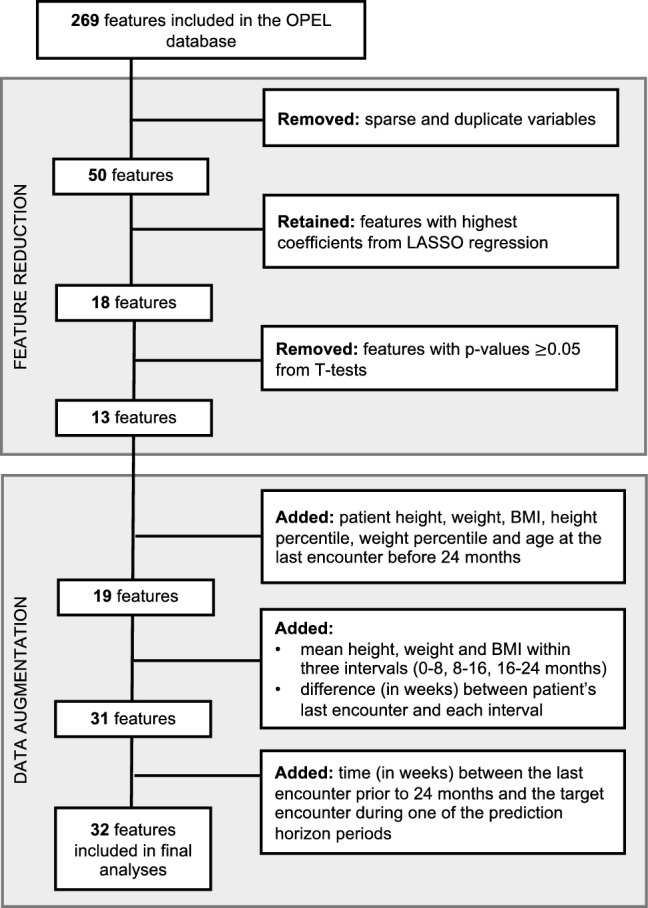
Feature reduction and data augmentation steps.

The proposed model was developed using a multi-stage process as summarized in Fig. 1, including: (1) raw data collection and integration; (2) data preprocessing; (3) feature engineering; and (4) model training and validation as described below.

### Raw data collection and integration

We extracted a subset of data from the Obesity Prediction in Early Life (OPEL) database, a longitudinal, EHR-based data repository that combines birth certificate, contextual-level, and health outcome data for 19,857 children born in Marion County, Indiana between 2004 and 2019. Linked data in the OPEL database are from three independent sources: (1) the Child Health Improvement through Computer Automation (CHICA) system, an electronic health record (EHR) and pediatric clinical decision support system that operated in 8 primary care clinics in Indianapolis between 2004 and 2019^27^; (2) the Indiana Standard Certificate of Live Birth (i.e., ‘birth certificate’), which was made available from the Marion County Public Health Department; and (3) the Social Assets and Vulnerabilities Indicators (SAVI) Project, a geocoded data repository^28^. Additional details of the independent data sources that comprise the OPEL database are described elsewhere^29^. Institutional Review Board approval for the OPEL data linkage and related analyses was obtained from the Indiana University School of Medicine. This study complies with all relevant ethical regulations and its protocols were approved by the Indiana University School of Medicine Institutional Review Board. Informed consent was waived for this retrospective study as the data were already collected. We did not generate any new data for this study.

### Data preprocessing

**Figure 2:**
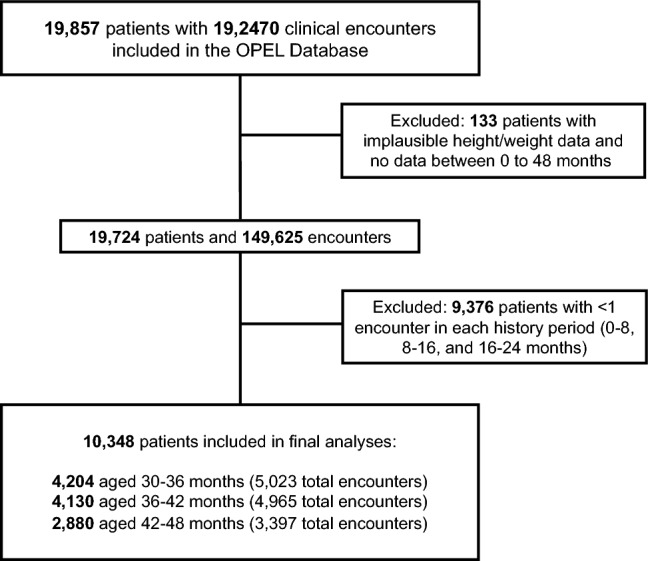
Participant flow.

The OPEL database contained 149,625 clinical encounters for 19,724 unique patients ages 0–48 months (Fig. 1). We performed data preprocessing to remove erroneous and implausible records, impute missing values, and scale numerical exposure variables to a range of [0, 1] (Fig. 2).

We set upper and lower limits for height and weight for each encounter using CDC growth charts^30^, categorizing values greater to or equal than three standard deviations from the mean as input error. We excluded records with erroneous height and weight since these variables were used to compute our outcome variable (BMI).

The available exposure variables were grouped into three categories for the purpose of imputation: mean imputation, historical imputation, and no imputation. Mean imputation was used for ordinal numerical variables such as weight, height, mother age, father age etc. For this group of variables, the missing value is replaced with the average of the values for the same variable from the nearest previous and next encounters. Historical imputation was used for variables that represent assessment results such as motor skills, as we assumed that in most cases, the previous assessment result was still valid unless otherwise indicated. We did not perform imputation for the remaining variables including whether the child has seen a dentist or had a recent oral exam, as imputation for these variables would not be meaningful.

### Feature engineering

The integrated dataset consisted of 269 variables (Fig. 1). Several of these variables were sparse and did not have sufficient data. Most of the sparse variables belonged to the no imputation group, but some were still sparse after imputation because a large number of the patients did not have values for these variables. In addition, some of the variables were duplicate. Eliminating sparse and duplicate variables resulted into 50 well-populated variables. We then applied least absolute shrinkage and selection operator (LASSO) regression and selected 18 features with the highest coefficients. We used the Q-Q plot to check for normality^31^. Subsequently, we conducted univariate T-tests and reduced the feature space to 13 features with p-values < 0.05. In addition to reducing the feature space, we used the T-test was to confirm that the initial cut-off threshold adopted in the LASSO selection captured all important features. The Wilcoxon Rank-Sum test was also considered as an alternative to the T-test given that both LASSO and T-test are both parametric statistical methods whereas the Wilcoxon Rank-Sum is non-parametric. However, since the dataset consists of a large number of samples, and the data processing step removed gross errors, the T-test is asymptotically valid and was preferred^32^.

The above group of features were directly obtained from the raw data. Following peer-reviewed literature on early life obesity risk (e.g., Ref.^23^), we augmented the feature space with a set of historical exposure variables derived from the patient’s weight, height and BMI at different time periods. The patients’ encounters were aggregated over three history periods: 0–8 months, 8–16 months, and 16–24 months of age. Patients without at least one clinical encounter in all three periods were excluded. For these patients, the proposed model would not have sufficient data to make any prediction. Moreover, this requirement is in line with clinical practice which recommends that children below the age of 24 months have at least one visit very 6 months^33^.

For each historical period, the mean height, mean weight, and mean BMI was calculated and added as separate exposure variables. In addition, the difference in patient age (in weeks) during each of these periods and the last clinical encounter recorded for the patient prior to 24 months of age were also obtained. These variables are used to temporally anchor the weight, height and BMI values from different historical period. Independently, the height, weight, BMI, height percentile, and weight percentile at 24 months (the end of the historical period) were also added as exposure variables. In summary, 19 features were added as the result of the above data augmentation procedure (Fig. 1). They consisted of:

- Mean height, weight and BMI during the periods 0–8, 8–16 and 26–24 months (9 variables).
- Historical time difference (in weeks) between the last encounter within three periods (0–8, 8–16, and 16–24 months) and the last encounter prior to 24 months (3 variables).
- Mean height, weight, BMI, height percentile, weight percentile and age at 24 months (6 variables).
- Prediction time difference (in weeks) between the last encounter prior to 24 months and the target encounter during one of the prediction horizon periods, as described next (1 variable).

Since the proposed model relies on a supervised learning methodology, outcome variables representing actual BMI values at each target encounter are needed for training as well as validation of the models. Moreover, to investigate the effect of the prediction horizon on the accuracy of the BMI value prediction, three prediction periods were considered: between 30 and 36 months, between 36 and 42 months, and between 42 and 48 months of age. It should be noted that the first prediction period has a gap of 6 months compared to the last historical period ending at 24 months. Sample records are constructed for each patient encounter within the prediction time periods. If there were multiple encounters for the same patient during any of the prediction periods, most of the exposure variables will be the same since they were collected prior to 24 months. The exception is the prediction time difference exposure variable mentioned above which indicates that the target BMI value is for a different patient’s age within the prediction horizon.

### Model training and validation

For each patient, the encounters were split by patient age, creating a history dataset (i.e., before 24 months of age) and a prediction dataset (i.e., after 24 months of age). Our outcome of interest was BMI according to patient age, in months, and sex, as defined by the Center for Disease Control and Prevention (CDC) guidelines^30^. We developed three models using the historical dataset, which contained data from the first 1000 days, to predict BMI at various ages: between ages 30–36 months, between ages 36–42 months, and between 42 and 48 months. All three models used the same feature values with the exception of the prediction time difference.

For all three models, we split the data into train (80% of the data) and validation (20% of the data) datasets for each fold of the fivefold cross validation. The split was performed at the patient level to ensure that patients did not participate in both training and validation. A random split based on geographic location was also considered; however, the data available was not sufficient.

All models were trained using support vector regression (SVR)^34^ with fivefold cross validation and a radial basis function (RBF) kernel. The model parameters gamma (the radial basis function variance) and C (margin) were optimized using the grid search technique over five values for each of the two parameters. The best models were obtained when gamma = 0.0001 and C = 1000. These parameters defined a SVR with a narrow margin and a high penalty for samples within the margin. The SVR transformation kernel is non-linear, therefore the coefficients of the regression in the model architecture are not directly related to the exposure variables. That said, the parameters described above, the list of exposure variables, and the training methodology completely define the proposed models.

We selected SVR due to its efficiency, simplicity, and ability to predict BMI trajectories. This machine learning technique in combination with the added temporal variables obtained from the data augmentation process enabled the models to use not only the instantaneous values of the exposure variables but also the progression of these variables over the patient’s history (e.g., mean weight during 0–8, 8–16 and 16–24 months).

Finally, once the models were trained with the 13 features extracted from the original dataset and the 19 features obtained from the augmentation process, we compared predicted patient BMI values generated by each of the models to patient BMIs recorded in the EHR for the three prediction horizons. This validation procedure is similar to the procedure needed to apply the models to new patients. First, the values of the exposure variables for each new patient have to be collected for the three observations periods 0–8 months, 8–16 months and 16–24 months. Second, the desired prediction horizon has to be selected (i.e., 30–36 months, 36–42 months, or 42–48 months) and the corresponding model is then used to compute the BMI for the selected horizon using the historical input data collected for the patient. Each model is an estimator of the BMI function. This estimator consists of a set of support vectors (i.e., a subset of the training samples), a kernel, and Lagrange multipliers which optimally represent the training data. In this study, the RBF kernel was selected. The vector of exposure variables for the new patient is transformed using RBF. The dot product of the resulting vector and the RBF of each of the support vector is then computed. Finally, each dot product is multiplied by the corresponding Lagrange multiplier and all are summed to produce the estimated BMI for the patient.

### Ethics declarations

Institutional Review Board approval for the OPEL data linkage and related analyses was obtained from the Indiana University School of Medicine. All methods were carried out in accordance with relevant guidelines and regulations.

## Results

**Figure 3:**
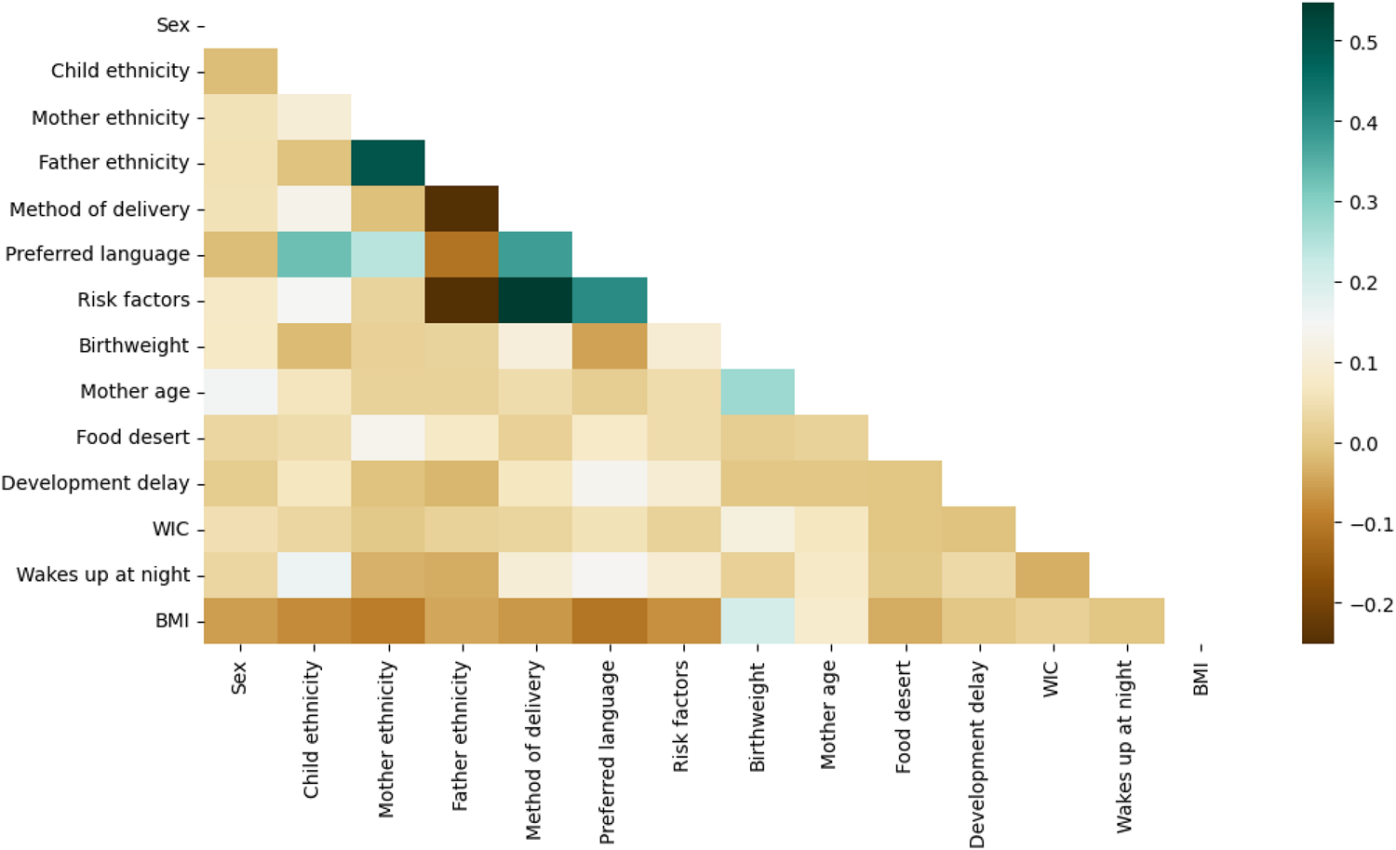
Correlation heatmap between the predictors included in the final models.

Figure 3 presents a correlation heatmap between the predictors included in the final models and Table 1 presents the correlations of the predictors with mean child BMI across the three prediction ranges. Table 1 reveals that most of the exposure variables in our model showed a significant correlation with child BMI across all three predictor ranges. For some of the variables (e.g., whether or not the child has ever been enrolled in WIC; whether or not the child had been diagnosed with a developmental delay; or whether the child wakes up at night and needs help going back to sleep), the correlation became more important at later prediction ages; whereas for others, the correlation appeared to be equally important for all three prediction ages (e.g., percent of population living in a food desert).

**Table 1:** List of top exposure variables from the raw data identified by LASSO and corresponding p-values for average BMI values for each prediction period obtained from univariate T-tests. The split criterion for each variable is indicated in the first column and the percentage of patients in each group is shown () below each p-value. The second column describes whether the variable is predictive, protective or inconclusive of an increase in BMI. The variables with inconclusive t-tests were excluded from the model (bottom 5 rows of the table). Numerical variables are indicated with *. All other variables are categorical.

Top LASSO variables	Relationship to BMI	Prediction age (months)		
		30–36	36–42	42–48
Total number of patients		4204	4130	2880
Selected features				
Child sex: male vs. female	Predictive	< 0.01 (51%/49%)	< 0.01 (52%/48%)	< 0.01 (53%/47%)
Hispanic ethnicity: yes vs. no				
Child	Predictive	< 0.01 (47%/53%)	< 0.01 (50%/50%)	< 0.01 (46%/54%)
Mother	Predictive	< 0.01 (45%/55%)	< 0.01 (48%/52%)	< 0.01 (45%/55%)
Father	Predictive	< 0.01 (64%/36%)	< 0.01 (66%/34%)	< 0.01 (63%/37%)
Method of delivery: cesarean/vaginal	Predictive	< 0.01 (28%/72%)	< 0.01 (27%/73%)	0.03 (28%/72%)
Parent’s preferred language: Spanish/English	Predictive	< 0.01 (35%/65%)	< 0.01 (37%/63%)	< 0.01 (35%/65%)
Maternal risk factors during pregnancy: Any/None	Predictive	< 0.01 (40%/60%)	< 0.01 (41%/59%)	0.02 (43%/57%)
*Child’s birthweight (g): > 4000 vs. ≤ 4000	Predictive	< 0.01 (11%/89%)	< 0.01 (10%/90%)	< 0.01 (12%/88%)
*Mother’s age when the child was born (years): > 30 vs. ≤ 30	Predictive	< 0.01 (28%/72%)	< 0.01 (28%/72%)	< 0.01 (27%/73%)
*Percent of population living in a food desert: mean < 32%	Protective	0.01 (27%/73%)	< 0.01 (28%/72%)	0.01 (27%/73%)
Whether the child has a developmental delay Suspected delay/typical development	Inconclusive	0.69 (16%/78%)	0.87 (17%/79%)	0.85 (13%/80%)
Diagnosed/suspected delay	Predictive	0.31 (6%/16%)	0.54 (4%/17%)	0.03 (7%/13%)
The child has ever been enrolled in WIC: Y/N	Predictive	0.19 (84%/16%)	0.08 (85%/15%)	0.03 (86%/14%)
Child wakes up at night and needs help going back to sleep: Y/N	Predictive	0.02 (70%/30%)	< 0.01 (66%/34%)	< 0.01 (63%/37%)
Excluded features				
The parent thinks the child has a sleeping problem: Y/N	Inconclusive	0.76 (10%/90%)	0.97 (10%/90%)	0.82 (11%/89%)
Parent is confident completing health forms: Y/N	Inconclusive	0.61 (6%/94%)	0.61 (6%/94%)	0.19 (6%/94%)
Parent is at risk for low health literacy: Y/N	Inconclusive	0.11 (47%/53%)	0.47 (51%/49%)	0.82 (46%/54%)
Parent reports doors at home are secure (e.g., child safety): Y/N	Inconclusive	0.42 (6%/94%)	0.81 (6%/94%)	0.92 (6%/94%)
*Child’s blood lead level: > 10 mg/dl vs. ≤ 10 mg/dl	Inconclusive	0.15 (8%/92%)	0.94 (8%/92%)	0.49 (9%/91%)

Table 1 also shows that children with higher birthweights, whose mothers were older than 30 years of age at delivery or who had any medical risk factors during pregnancy (e.g., a flag for any one of the 13 possible medical risk factors reported on the birth certificate, including antiretroviral therapy, gestational diabetes, pre-pregnancy/chronic diabetes, and eclampsia), with parents of Hispanic ethnicity, and who experienced nighttime wakings and needed help going back to sleep had higher average BMIs across all three prediction ranges. Conversely, living in a food desert was protective of BMI. All of these variables, regardless of whether they had a significant correlation with average BMI during early, late, or all prediction age ranges were retained as input features in the proposed model.

Variables that did not show significant correlations with child BMI were not retained in the proposed prediction model. These variables, listed at the bottom of Table 1, included those assessing whether parents believe their child had a sleeping problem, parental health literacy, safety at home, and child blood lead level. Thus, from the initial 19 variables identified using Lasso, we retained 13 based on the results of the T-tests.

In addition to the variables that were directly derived from the OPEL database discussed above, we included child sex and several history variables derived from children’s weight and height (Fig. 1). These included height, weight, and BMI at the end of the history period (i.e., 24 months), as well as average height, weight, and BMI over three stratas of the history period (0–8, 8–16 and 16–24 months). We preformed the T-test for each of these derived variables over the three prediction age ranges with the mean value of the variable as the split value to confirm their predictive potential. For the sex variable, the split was performed based on male versus female. The p-values (Table 2) indicate that each of these variables had a significant, positive correlation with the child’s average BMI. A noted exception is the relationship between the average height in the history period between 0 to 8 months, for which the correlation with child average BMI between 42 and 48 months was not statistically significant.

**Table 2:** List of historical exposure variables obtained from data augmentation and derived from the height, weight and BMI of the patient. All the variables are continuous. Table shows p-values for average BMI values for each prediction period using univariate T-tests. The mean value was used for the cohort split for all variables. The number of patients for each time period is shown in the first row and the percentage of patients in each group is shown below the p-value.

	Prediction age, months		
	30–36	36–42	42–48
Number of patients	4204	4130	2880
Height			
At 24 months	< 0.01 (50%/50%)	< 0.01 (51%/49%)	< 0.01 (50%/50%)
Percentile, 24 months	< 0.01 (51%/49%)	< 0.01 (51%/49%)	< 0.01 (50%/50%)
Mean, 0–8 months	< 0.01 (54%/46%)	< 0.01 (52%/48%)	0.12 (50%/50%)
Mean, 8–16 months	< 0.01 (52%/48%)	< 0.01 (52%/48%)	< 0.01 (51%/49%)
Mean, 16–24 months	< 0.01 (49%/51%)	< 0.01 (49%/51%)	< 0.01 (49%/51%)
Weight			
At 24 months	< 0.01 (54%/46%)	< 0.01 (54%/46%)	< 0.01 (55%/45%)
Percentile, 24 months	< 0.01 (51%/49%)	< 0.01 (50%/50%)	< 0.01 (51%/49%)
Mean, 0–8 months	< 0.01 (49%/51%)	< 0.01 (49%/51%)	< 0.01 (48%/52%)
Mean, 8–16 months	< 0.01 (50%/50%)	< 0.01 (50%/50%)	< 0.01 (52%/48%)
Mean, 16–24 months	< 0.01 (53%/47%)	< 0.01 (52%/48%)	< 0.01 (52%/48%)
BMI			
At 24 months	< 0.01 (53%/47%)	< 0.01 (53%/47%)	< 0.01 (53%/47%)
Mean, 0–8 months	< 0.01 (53%/47%)	< 0.01 (53%/47%)	< 0.01 (51%/49%)
Mean, 8–16 months	< 0.01 (53%/47%)	< 0.01 (53%/47%)	< 0.01 (50%/50%)
Mean, 16–24 months	< 0.01 (53%/47%)	< 0.01 (53%/47%)	< 0.01 (52%/48%)

We observed significant missing data and attrition, both in terms of patients who did not have data from enough encounters in the history dataset, and patients with sufficient history data who did not have any recorded encounters in the prediction dataset. Figure 2 presents the number of unique patients who had at least one clinical encounter recorded within each of the history periods and one clinical encounter for each respective prediction period (i.e., every 6 months). This is consistent with clinical recommendations for pediatric well child visits^35^.

Table 3 shows the performance of the three models for each prediction period, reported as the mean average error (MAE), its standard deviation, and the 95% confidence interval (CI) over the fivefold cross-validation. The results in Table 3 indicate that the models are able to predict the BMI of each patient with a high level of accuracy and a narrow confidence interval. Deviations between the predicted and actual BMI values increase with increasing prediction horizons.

**Table 3:** Model performance indicators for each prediction period.

Prediction period	BMI, mean (SD)	MAE (SD)	95% CI
30–36 months	16.8 (1.95)	0.96 (0.02)	(0.93–1.06)
36–42 months	16.7 (1.93)	0.98 (0.03)	(0.95–1.08)
42–48 months	16.6 (1.86)	1.00 (0.02)	(0.94–1.10)

**Figure 4:**
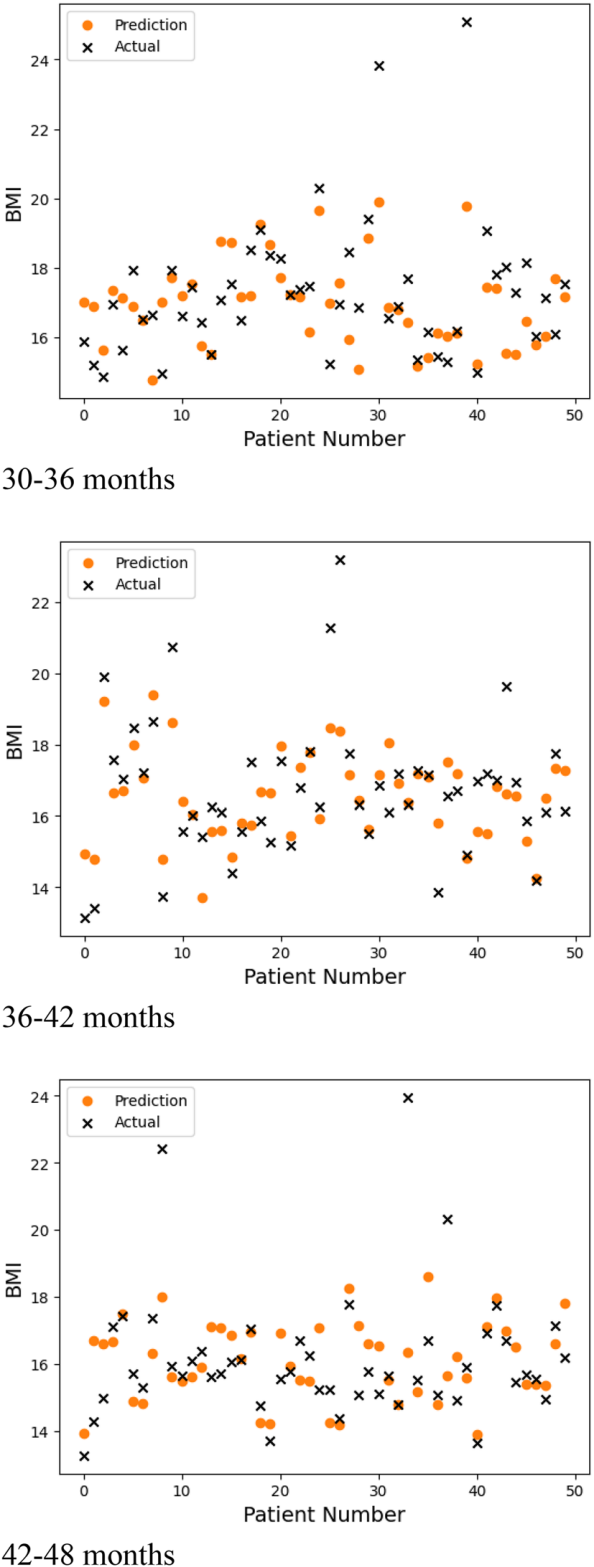
Predicted and recorded BMI values for a sample of randomly selected patients (n = 50) at 30–36 months, 36–42 months, and 42–48 months of age.

**Figure 5:**
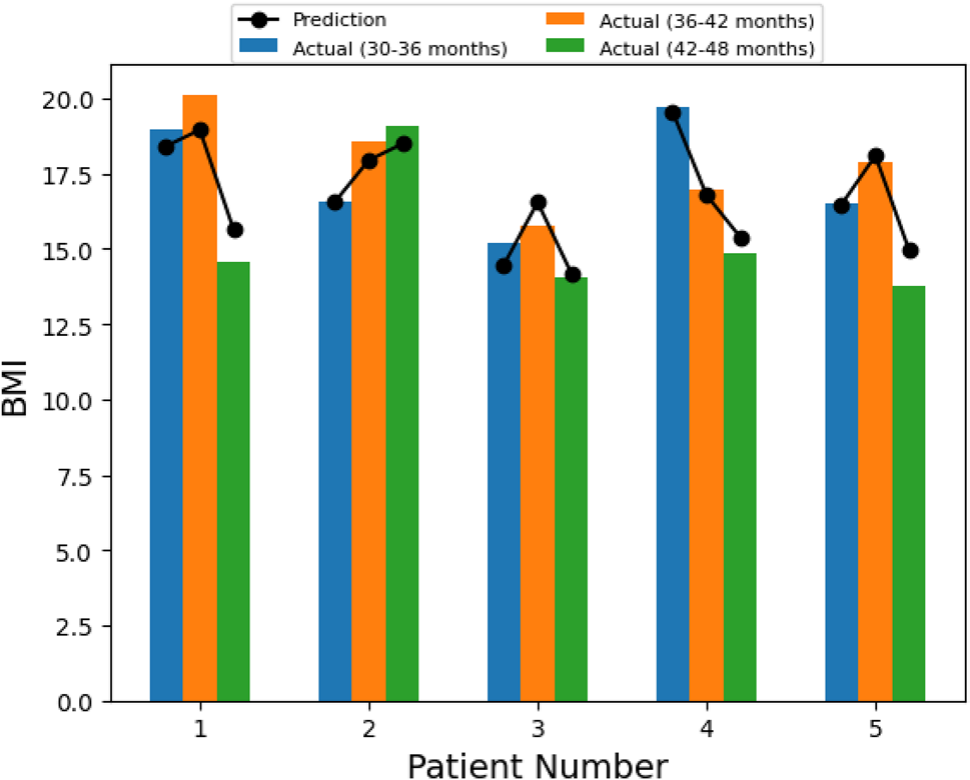
Sample BMI prediction trajectory for 5 selected patients.

Figure 4 displays the predicted BMIs generated by each of the models (i.e., at 30–36 months, 36–42 months, and 42–48 months) and the recorded BMI values during the same time period for a random sample of 50 patients. These figures indicate that the models accurately tracked BMI values for each patient. Figure 5 displays the BMI trajectories predicted by our models for a random sample of 5 patients over the three prediction periods. The models were able to accurately predict both upward and downward changes in BMI.

## Discussion

Despite epidemiological evidence of modifiable risk factors for obesity and calls for early intervention, few existing obesity prevention trials focus on children younger than 5 years old^19,36^. In this study, we used machine learning algorithms to identify children with high risk for developing obesity that could be targeted for intervention. Using LASSO and support vector regression, we predicted children’s BMI between ages two and four using factors from the first 1000 days that were available in a unique, longitudinal, population-based dataset that combines EHR, birth certificate, and geocoded data.

Our models have relevance for clinical and public health efforts to prevent unhealthy weight gain and obesity in early childhood. First, unlike other models that predict children’s BMI or weight thresholds at a single point in time, our models predict BMI in three future 6 month intervals (i.e., 30–36 months, 36–42 months, and 42–48 months). This may be beneficial from a clinical perspective, as it would enable pediatric providers to observe potential BMI increases over an extended period. As such, health care providers could use the models to design intervention plans that address modifiable risk factors more applicable to different age groups.

Another added benefit to providing health care providers with estimated future BMI values is that it might aid in discussions about preventive measures to reduce children’s obesity risk. The American Academy of Pediatrics (AAP) recommends measuring BMI and screening for obesity-related comorbidities as part of routine primary care beginning at age two, but research indicates that parents^37^ and providers^38,39^ often avoid the subject. In turn, parents who have inaccurate perceptions of their child’s obesity risk are more likely to ignore appropriate health messages^40^. Anticipatory guidance around obesity is not part of standard care prior to age two, despite evidence of modifiable risk factors^23^. One reason why obesity counseling in infancy may be challenging is because parents may be unaware of the importance of prevention. A dynamic, predictive BMI tracker, like the one we propose here, could alert providers of children with high risk of developing obesity prior to the onset of unhealthy weight, and help frame conversations about obesity prevention and risk. This may be more effective in prompting behavioral change than evaluating and counseling based on risk factors alone, as other work shows that that patients’ understanding of their own risk is a key first step in the process of behavior change^41^.

The present study also highlights the importance of several risk factors for increased BMI in the first 1000 days. Consistent with epidemiological research^23,42^, we identified several potentially modifiable factors associated with higher child BMI in both the prenatal and early infancy periods, including maternal risk factors during pregnancy, cesarean delivery, higher infant birthweights, and whether the child wakes up at night and needs help falling back asleep. Other factors, like the percentage of the population living in a food desert, were protective of increased BMI, which conflicts with some^43^, but not all, published literature^44,45^. It is possible that our food desert variable was confounded by other, unmeasured factors. Children’s address data were also extracted from the birth certificate, so we were unable to account for residential movement. In addition, while many markers of socioeconomic risk, including Hispanic ethnicity, Spanish parent language preference, and being enrolled in WIC were also predictive of higher BMIs, others, like low health literacy and children’s blood lead levels, were not associated with a significant increase in BMI. While our model and methods should be tested in other populations, our analysis provides important proof of concept that children’s BMI trajectories can be predicted by modifiable risk factors in early life and thus lend support to efforts to intervene prior to the onset of unhealthy weight gain. Our model supports other research suggesting that improving women’s health during pregnancy, improving children’s sleep, and alleviating socioeconomic risk may have important downstream impacts on children’s weight trajectories in later childhood^23^.

Our study addresses several limitations of existing studies focused on using machine learning to predict obesity in early childhood. First, most previous models of childhood obesity risk focus their prediction horizons after age five. Our models could provide health care providers with BMI values over three consecutive prediction periods between 2 and 4 years of age, a critical window where interventions to prevent obesity may be more successful than those targeting weight loss in older populations. Second, existing models often classify patients according to a pre-specified threshold of obesity risk^46–48^, which poses some limitations. For example, binary classifications of risk are not always explainable, and a slight deviation in the exposure variables may result in a sudden change in the outcome from at risk to no risk or vice-a-versa. By predicting BMI values, our proposed models consider the underlying growth trajectory for both weight and height instead of focusing only on the weight of the patient. Finally, the proposed models are cost-effective since they primarily depend on EHR data. We acknowledge that some of the sociodemographic variables available in CHICA are not routinely collected as part of the clinical health record, and that EHR data typically contain information on maternal prenatal risk factors separately from risk factors during infancy and from measures of height and weight across childhood. However, the limited number of factors we identified can be easily collected from patients using existing screeners, facilitating BMI prediction in real time. This is an improvement over existing models that rely on survey data, which may incur significant additional cost and have limited applicability to a wider pediatric population.

We observed a slight decrease in the accuracy of BMI prediction at older ages, such that predictions were more accurate for the 30–36 month prediction period compared with the 42–48 month period. This is expected since all three models use the same history period (0–24 months). We hypothesize that a significant improvement in the 36–42 months and 42–48 months prediction period models would have been observed if these models used more recent history data. Our history period was limited to 0–24 months in order to evaluate the importance of risk factors in the first 1000 days and the potential for the models to predict future BMI within three 6 month intervals. As displayed in Table 3, we were able to achieve this with a high level of confidence. Future work should model a more granular trajectory of BMI across early childhood, which we were unable to do with our selected methods and available data.

Our study is subject to some limitations. First, maternal risk factors during pregnancy is a composite variable flagging the presence of at least one of 13 possible medical conditions that appear on the birth certificate, including those with established relationships to childhood obesity (e.g., diabetes and gestational hypertensive disorders)^49,50^ and those that have been underexplored. We were unable to determine whether certain maternal risk factors influenced children’s BMIs more than others. Some of our data come from parental report, which may be subjective. Our study population, which is from a single county in central Indiana, is not necessarily representative of broader populations. An external validation from a different population would be useful to confirm the generalizability of current findings. Future studies incorporating bigger cohorts from different populations would allow an assessment of the generalizability of the proposed models and could improve their performance.

## Conclusion

In this study, we predicted with reasonable accuracy children’s BMI between ages two to four years using risk factors from the first 1000 days of life. By leveraging a linked dataset combining maternal and child data across prenatal and early life, we were able to highlight modifiable risk factors for increased BMI during these critical periods of the life course. While the proposed model is predictive rather than causal, several of the important predictive variables of the model align with findings from prior research which indicates that improving women’s health during pregnancy, improving children’s sleep, and alleviating socioeconomic risk may have important downstream impacts on children’s weight trajectories in later childhood. Moreover, by providing predicted BMI values, our models might also facilitate conversations between providers and parents about obesity risk and prevention in early life and enable clinicians to identify children with higher future obesity risk, enabling early intervention.

## Data Availability

The datasets used and/or analyzed during the current study and the associated models are available from the corresponding author upon reasonable request.

## Abbreviations

BMI: Body Mass Index
CHICA: The child health improvement through computer automation system
EHR: Electronic health record
LASSO: Least absolute shrinkage and selection operator
SVR: Support vector regression
RBF: Radial basis function
WIC: Special supplemental nutrition program for women, infants, and children
MAE: Mean average error
CI: Confidence interval
